# Effect of intraoperative operating table rotation on lower limb perfusion index in patients in the lithotomy position

**DOI:** 10.1097/MD.0000000000030677

**Published:** 2022-09-23

**Authors:** Kentaro Hara, Kodai Ichihara, Michiko Yamaguchi, Hiroaki Takeshita, Tamotsu Kuroki

**Affiliations:** a National Hospital Organization Nagasaki Medical Center, Nagasaki, Japan; b Nagasaki University Graduate School of Biomedical Sciences, Nagasaki, Japan.

**Keywords:** limb perfusion index, lithotomy position, observational study, operating table rotation, returning to the horizontal position

## Abstract

We focused on “returning to the horizontal position,” one of the measures for preventing well leg compartment syndrome implemented at our hospital, and aimed to clarify the effect of intraoperative positional changes by operating table rotation on blood perfusion in the lower extremities during lithotomy in patients under general anesthesia. This prospective observational study examined 64 patients scheduled to undergo general anesthesia in the lithotomy position from March 2021 to May 2022. The primary endpoint was the perfusion index (PI) of the lower limb before and after operating table rotation. The baseline lower limb PI before the operating table rotation was 2.376 (1.591), and the lower limb PI after the change from Trendelenburg to the horizontal position was as follows: immediately after, 2.123 (1.405); 5 minutes, 1.894 (1.138); 10 minutes, 1.915 (1.167); and 15 minutes, 1.993 (1.218). Compared with the baseline, no significant difference was noted in the change in the lower limb PI due to the Trendelenburg to horizontal positional change. The baseline lower leg pressure before the operating table rotation was 51.4 (13.4) mm Hg, and the lower leg pressure after the change from the Trendelenburg to the horizontal position was as follows: immediately after, 36.6 (10.3) mm Hg; 5 minutes, 36.5 (10.2) mm Hg; 10 minutes, 36.4 (10.0) mm Hg; and 15 minutes, 36.5 (10.2) mm Hg. Compared with the baseline, the change in lower leg pressure due to the Trendelenburg to horizontal positional change showed a significant decrease immediately afterward (*P* < .001). After operating table rotation from the Trendelenburg to the horizontal position, the lower limb PI did not change significantly after 15 min. However, lower leg pressure showed a significant decrease immediately after returning to the horizontal position. This result provides evidence for operating table rotation as a preventive measure for well leg compartment syndrome.

## 1. Introduction

Patients undergoing surgery should be optimally fixed in the surgical position based on the surgical site, procedure, and anesthesia method. However, patients are exposed to various postoperative complications related to the surgical position. One important complication related to surgical position is compartment syndrome after lithotomy, wherein the pressure inside the muscle compartment covered by fascia is increased due to conditions such as edema and hemorrhage, resulting in microcirculatory disturbances innervating the muscles and nerves. In 1979, Leff et al^[1]^ reported compartment syndrome of the lower leg after total cystectomy and urethroplasty performed in the lithotomy position, and this condition has been termed “well leg compartment syndrome” (WLCS). The incidence of WLCS has been reported by Halliwill et al^[2]^ over the past 30 years; data for the lithotomy position showed a frequency of 1 in 3500 cases. Simms and Terry^[3]^ reported a WLCS incidence of 1 in 500 cases of total bladder cystectomy, and cases have also been reported in surgery and obstetrics and gynecology.^[4–7]^ WLCS can cause critical sequelae in patients, including metabolic acidosis, atrophic renal failure, Volkmann contracture, limb loss, and death.^[8]^ Risk factors for WLCS have been identified as an operative time longer than 4 hours, lithotripsy, peripheral vascular disease, obesity, diabetes, use of intermittent pneumatic compression devices, insufficient intravascular volume, traction, and compression of blood vessels by pelvic manipulation, and compression of the lower extremities.^[8]^

At our surgical center, WLCS has been reported in 0.83% of cases.^[9]^ Therefore, we consulted operating room nurses, general surgeons, obstetricians, and urologists and developed the following recommendations for procedures that can be modified to the open leg position: a-1) change from the lithotomy position to the open leg position; b-1) base lower limb elevation restriction on the right atrium height; b-2) prevention of compression at the lower leg contact site; and b-3) provision of nursing care. Five interventions completely prevented the incidence of WLCS, including a-2) intraoperative decompression of the lower leg contact site by the surgeon and b-4) returning the patient to the horizontal position every 3 hours by Trendelenburg position.^[9]^ No occurrence of WLCS has been reported at our operation center since these measures were implemented. However, the lack of supporting evidence, such as changes in blood perfusion due to interventions, remains an issue for further investigation. Modifications in blood perfusion of the body can be observed by rotating the operating table and changing the patient’s position.^[10,11]^ However, most previous studies on this topic have been conducted with unanesthetized healthy adults, and to the best of our knowledge, no studies have reported on blood flow changes caused by operating table rotation in patients under general anesthesia. Among the regional studies, there are only reports on the blood perfusion index (PI) in the lower limbs of neonates.^[12]^

Therefore, we focused on “returning to the horizontal position,” which is one of the WLCS prevention measures implemented at our hospital, to clarify the effect of intraoperative positional changes due to operating table rotation on blood perfusion in the upper and lower extremities during lithotomy in patients under general anesthesia. We hypothesized that the PI of the lower limb would increase as a result of returning the patient from the Trendelenburg to the horizontal position. The results of this study are expected to provide evidence for WLCS prevention measures.

## 2. Methods

### 2.1. Ethical considerations

This study was approved by the Ethics Committee of the Nagasaki Medical Center (approval number: 2021009). The researchers provided written and oral explanations to the study participants about the study procedures that were approved by the head of the research institution. The participants were also provided with opportunities to ask questions and sufficient time to decide whether to provide their consent to participate. For participation in the study and publication of the results, all participants provided written informed consent of their free will after confirming that they understood the study.

### 2.2. Study design, setting, and population

This prospective observational study of patients scheduled to undergo general anesthesia in the lithotomy position at the Nagasaki Medical Center Surgical Center was conducted from March 2021 to May 2022 (patient enrollment period: March–August 2021 and February–April 2022). The exclusion criteria were emergency surgery and pediatric patients.

### 2.3. Data collection and processing

The following clinical information of the study participants was obtained from their medical records: patient information included age, sex, height, weight, body mass index, and medical history (presence of hypertension, peripheral circulatory disorders, and ischemic diseases). Surgical information included the surgical time and lower leg pressure. The operating table rotation in this study indicates a positional change from the Trendelenburg to the horizontal position. The lower leg pressure measurement site was the lower leg of the lower extremity, which became the upper leg when the head was lowered. Anesthesia information included American Society of Anesthesiologists classification, anesthesia duration, infusion volume, urine output, intraoperative vital signs (systolic blood pressure [BP], diastolic BP, and pulse rate), and PI. For PI during Trendelenburg to horizontal position change, data from the timing of Trendelenburg to horizontal position return, which was performed 3 h after the start of surgery, were used.

### 2.4. Equipment

PI is expressed as the ratio of pulsatile to nonpulsatile blood volume in peripheral tissues. PI is a noninvasive measure of peripheral perfusion that can be measured continuously and noninvasively using a pulse oximeter (range: 02–20%).^[13,14]^ Because the PI of the toe as a local PI correlates with blood flow in the triceps^[12]^ and the upper limb PI is used as an index of systemic circulatory dynamics in patients under general anesthesia,^[15]^ we decided to obtain the lower limb PI. A Radical-7® Pulse CO-Oximeter® (Masimo) was used. Intraoperative body pressure: The SR Soft Vision software (Sumitomo Science and Technology) was used. The measurement range was 20–200 mm Hg, and the data were saved as continuous data in a video format. Operating table and lithotripter: The operating table used was Operating Table MOT-5701® (Mizuho), and the lithotripter used was Levitator® (Mizuho).

### 2.5. Primary and secondary endpoints

The primary endpoint was the PI of the lower limb before and after rotation of the operating table. Baseline data were collected 1 min before rotation and continuously from immediately after to 15 min after rotation. Fifteen minutes after the rotation was set based on the progress of surgery at our institution, and because it is the time when blood flow stabilizes after the positional change.^[16]^ The lower extremity PI measurement site was the second toe of the lower limb that is on the upper side when the head is lowered.

The secondary endpoint was the change in leg pressure before and after operating table rotation from Trendelenburg to the horizontal position.

### 2.6. Statistical analysis

The study was set up with an effect size of.3, a significance level of.05, and a power of.8, calculated with G*Power 3.1.9.4 (Heinrich-Heine Universität), resulting in 64 patients. Simple tabulations were performed for patient, surgical, and anesthesia information, and the means (standard deviations) are shown.

**Figure 1.**
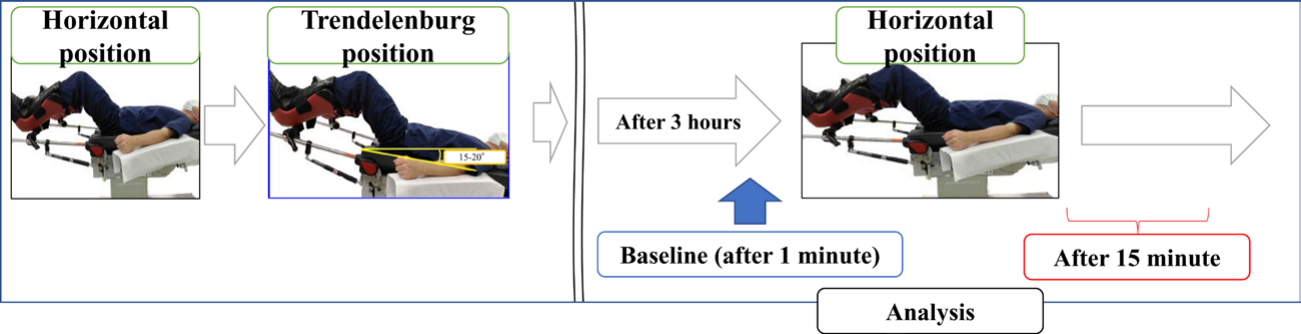
Flowchart of statistical analysis. The baseline was 1 min before the return to the horizontal position, and the data 15 min after the return to the horizontal position were analyzed.

PI and leg pressure data were collected continuously from the operating table rotation baseline for 15 min; however, data collected every 5 min were used for statistical analysis. To minimize potential bias and accuracy issues, we used the mean of 10 s of data before and after the rotation (a total of 11 data points from 50 s to 1 min and 10 s for the 1-minute data). The mean values from immediately after the operating table was rotated 15 min later, and the mean (standard deviation) of the change value and rate of change compared with 1 min before and 15 min after rotation were obtained (Fig. 1).

For the primary endpoint, lower limb PI data were collected every 5 min up to 15 min after the rotation of the operating table (baseline and immediately after, baseline and 5 min after, baseline and 10 min after, and baseline and 15 min after). If normality was present, the paired *t* test was used to analyze the data; if normality was absent, the Wilcoxon signed-rank test was used. The Shapiro–Wilk test was used to test normality.

For the secondary endpoint, that is, leg pressure changes before and after rotation of the operating table, data were collected every 5 min up to 15 min (baseline and immediately after, baseline and 5 min after, baseline and 10 min after, and baseline and 15 min after) from immediately after the rotation of the operating table. If normality was found, the paired *t* test was used to analyze the data; if there was no normality, the Wilcoxon signed-rank test was used.

Differences were considered significant at the 5% significance level. The JMP® 16 software (SAS Institute Inc., Cary, NC) was used for all statistical analyses.

## 3. Results

### 3.1. Patient attributes

Among the 70 patients who participated during the study period, 6 were excluded due to emergency surgery (n = 4) and consent was not obtained (n = 2). Ultimately, 64 patients were included in the analysis. The patient background characteristics and vital signs are shown in Tables 1 and 2, respectively.

**Table 1:** Patient characteristics (N = 64).

Age (yr)	68.5 (12.9)
Sex	
Male	36 (56.3%)
Female	28 (43.7%)
Height (cm)	160.7 (10.5)
Weight (kg)	59.6 (14.7)
Body mass index (kg/m^2^)	22.8 (4.4)
Anesthesia time (min)	436.3 (103.2)
Operative time (min)	358.3 (103.7)
Volume of bleeding (mL)	41.3 (52.9)
Urine volume (mL)	361.9 (244.0)
Total fluid volume (mL)	2638.1 (661.5)
Preoperative total protein level (g/dL)	6.6 (0.8)
Preoperative albumin level (g/dL)	4.0 (0.8)
Preoperative hemoglobin level (g/dL)	11.9 (2.3)
Hypertension	
Yes	15 (23.4%)
No	49 (76.6%)
Diabetes mellitus	
Yes	3 (4.9%)
No	61 (95.1%)
Ischemic heart disease	
Yes	4 (6.3%)
No	60 (93.7%)
American Society of Anesthesiologists-physical status	
I	3 (4.7%)
II	60 (90.6%)
III	3 (4.7%)
Stage of cancer	
I	19 (29.7%)
II	23 (35.9%)
III	21 (32.8%)
IV	1 (1.6%)

Values are presented as mean (standard deviation) or number of patients (%).

**Table 2:** Changes in vital signs after the change from the Trendelenburg position to the horizontal position (N = 64).

	Systolic BP (mm Hg)	Diastolic BP (mm Hg)	Pulse rate (bpm)
Baseline	112.5 (17.0)	62.2 (10.5)	64.1 (8.5)
Immediately after	108.1 (19.7)	58.2 (11.4)	63.7 (8.3)
5 min	102.4 (16.1)	52.2 (8.9)	63.5 (8.7)
10 min	101.3 (14.8)	51.8 (9.0)	63.3 (9.2)
15 min	101.9 (14.2)	53.1 (9.8)	64.3 (8.4)

Values are presented as mean (standard deviation).

BP = blood pressure.

### 3.2. Comparison of change in lower limb PI after the Trendelenburg to horizontal positional change

The baseline lower limb PI before operating table rotation was 2.376 (1.591), and the lower limb PI after the change from Trendelenburg to the horizontal position was as follows: immediately after, 2.123 (1.405); 5 min, 1.894 (1.138); 10 min, 1.915 (1.167); and 15 min, 1.993 (1.218). Compared with baseline, no significant difference was noted in the change in lower limb PI after Trendelenburg to the horizontal positional change (Table 3).

**Table 3:** Changes in lower limb PI after the change from the Trendelenburg position to the horizontal position (N = 64)

	Lower limb finger PI	*P* value*
Baseline	2.376 (1.591)	–
Immediately after	2.123 (1.405)	.28
5 min	1.894 (1.138)	.05
10 min	1.915 (1.167)	.05
15 min	1.993 (1.218)	.10

Values are presented as mean (standard deviation).

PI = perfusion index.

* Baseline versus each lower limb PI (*P* < .05).

### 3.3. Comparison of change in the lower leg pressure after positional change

The baseline lower leg pressure before operating table rotation was 51.4 (13.4) mm Hg, and the lower leg pressure after the change from the Trendelenburg to the horizontal position was as follows: immediately after, 36.6 (10.3) mm Hg; 5 minutes, 36.5 (10.2) mm Hg; 10 minutes, 36.4 (10.0) mm Hg; and 15 minutes, 36.5 (10.2) mm Hg. Compared with baseline, the change in lower leg pressure after Trendelenburg to horizontal positional change showed a significant decrease immediately afterward (*P* < .001) (Table 4).

**Table 4:** Changes in lower leg pressure after the change from the Trendelenburg position to the horizontal position (N = 64)

	Body pressure in the lower limbs (mm Hg)	*P* value*
Baseline	51.4 (13.4)	
Immediately after	36.6 (10.3)	<.001*
5 min	36.5 (10.2)	<.001*
10 min	36.4 (10.0)	<.001*
15 min	36.5 (10.2)	<.001*

Values are presented as mean (standard deviation).

* Baseline versus each lower leg pressure (*P* < .05).

## 4. Discussion

Recently, many endoscopic surgical operations have been performed because minimally invasive surgery is required owing to the short hospital stay, less bleeding during surgery, and small surgical wounds. However, many endoscopic surgeries require rotation of operating tables to secure the surgical field. In a previous study, hemodynamic changes in upper limb PI elevation were observed when lower limb elevation was performed in an unanesthetized participant.^[11]^ In the present study, prospective observation of lower limb PI in patients undergoing operating table rotation under general anesthesia from the Trendelenburg to horizontal position showed no significant increase in the lower limb PI. Our initial hypothesis was that the PI of the lower limb increases as a result of the patient returning from the Trendelenburg position to the horizontal position. Cardiovascular factors that increase blood flow include increased cardiac output per beat, increased beating rate, decreased aortic wall extensibility, and increased peripheral vascular resistance.^[17]^ Diastolic BP is affected by heart rate and peripheral vascular resistance. Moreover, it has been reported that elevation of the lower extremities increases the amount of circulating blood in the heart.^[18]^ Diastolic BP is partly related to the volume of blood ejected; however, it is also greatly affected by peripheral vascular resistance, in particular by hemodynamics. The study results showed that leg pressure at the lithotomy table contact site decreased significantly immediately after operating table rotation from the Trendelenburg to the horizontal position. This may have decreased peripheral vascular resistance, contributing to the decrease in diastolic BP. Therefore, we speculate that the decrease in systolic and diastolic BP when the patient was moved from the Trendelenburg position to the horizontal position did not lead to a significant increase in the lower limb PI.

A previous study reported that approximately 15 min was needed for blood flow in the body to stabilize after positional change.^[16]^ However, in our study, after the Trendelenburg to horizontal positional change, the lower limb PI did not increase significantly after 15 min, indicating that hemodynamic changes did not occur during the rotation of the operating table in patients under general anesthesia.

The lower limb pressure significantly decreased immediately after returning to the horizontal position from the Trendelenburg position. Among our WLCS prevention measures, one of the interventions is performing a horizontal return every 3 hours to improve blood flow to the lower extremities.^[9]^ Although there was a decreasing trend in the lower limb PI, there was no significant difference in the PI when patients were returned to the horizontal position from the Trendelenburg position. However, a change in the lower leg pressure should be noted. Our study showed that lower leg pressure significantly decreased in the horizontal position. This result is consistent with the fact that the pressure in the lower leg changes after rotating the operating table.^[19]^ Lower leg pressure and the lithotomy position can be risk factors for WLCS.^[20,21]^ Thus, we speculate that the return from the Trendelenburg to the horizontal position is associated with local pressure release, although it does not seem to contribute to improved blood flow.

We found that returning to the horizontal position did not significantly increase the lower limb PI. Therefore, returning to the horizontal position to improve the blood flow and prevent WLCS is not reasonable. However, because the leg pressure decreased significantly immediately after returning to the horizontal position, it can be inferred that the horizontal position is associated with the prevention of WLCS from the perspective of releasing local pressure. In the present study, patients were returned to the horizontal position every 3 h; however, the timing and degree of leg compression that lead to the prevention of WLCS must be considered further.

The limitations and future challenges of this study are as follows: First, this was a single-center, single-case, prospective observational study; a more detailed analysis is warranted, with more case studies at multiple centers. Second, our study was limited to laparoscopic cases; lower limb PI changes in robotic surgery cases must be observed where the head is lowered by 25°–30°, owing to the recent social trend toward artificial intelligence and robot-assisted surgery. Third, it was found that the lower leg pressure increased by the Trendelenburg position could be reduced by the horizontal position, but it was not possible to identify unacceptable lower leg pressure as a risk factor for WLCS. In the future, we would like to clarify the set point of the lower leg pressure of WLCS generation by advancing the analysis of WLCS and lower leg pressure.

## 5. Conclusions

After operating table rotation from the Trendelenburg to the horizontal position, the lower limb PI did not increase significantly after 15 min. However, lower leg pressure showed a significant decrease immediately after returning to the horizontal position. This result provides evidence for operating table rotation as a preventive measure for WLCS. All lithotomy surgeries require a return from Trendelenburg to a horizontal position every 3 hours to reduce lower leg pressure.

## Acknowledgments

We gratefully acknowledge the work of past and present members of our medical center. We would like to thank Paperpal Preflight Service (https://preflight.paperpal.com/partner/wolterskluwer/medicine/submit) and Editage (https://www.editage.jp/) for English language editing.

## Author contributions

Kentaro Hara and Kodai Ichihara were responsible for the organization and coordination of the study. Kentaro Hara was the chief investigator responsible for the data analysis. Michiko Yamaguchi and Hiroaki Takeshita provided study data. Tamotsu Kuroki made critical revisions to incorporate relevant information. All authors contributed to the writing of the final manuscript and approved it.

**Conceptualization:** Hiroaki Takeshita, Kentaro Hara, Kodai Ichihara, Michiko Yamaguchi, Tamotsu Kuroki.

**Data curation:** Hiroaki Takeshita, Kentaro Hara, Kodai Ichihara, Michiko Yamaguchi, Tamotsu Kuroki.

**Formal analysis:** Hiroaki Takeshita, Kentaro Hara, Kodai Ichihara, Michiko Yamaguchi, Tamotsu Kuroki.

**Funding acquisition:** Hiroaki Takeshita, Kentaro Hara, Kodai Ichihara, Michiko Yamaguchi, Tamotsu Kuroki.

**Investigation:** Hiroaki Takeshita, Kentaro Hara, Kodai Ichihara, Michiko Yamaguchi, Tamotsu Kuroki.

**Methodology:** Hiroaki Takeshita, Kentaro Hara, Kodai Ichihara, Michiko Yamaguchi, Tamotsu Kuroki.

**Project administration:** Hiroaki Takeshita, Kentaro Hara, Kodai Ichihara, Michiko Yamaguchi, Tamotsu Kuroki.

**Resources:** Hiroaki Takeshita, Kentaro Hara, Kodai Ichihara, Michiko Yamaguchi, Tamotsu Kuroki.

**Software:** Hiroaki Takeshita, Kentaro Hara, Kodai Ichihara, Michiko Yamaguchi, Tamotsu Kuroki.

**Supervision:** Hiroaki Takeshita, Kentaro Hara, Kodai Ichihara, Michiko Yamaguchi, Tamotsu Kuroki.

**Validation:** Hiroaki Takeshita, Kentaro Hara, Kodai Ichihara, Michiko Yamaguchi, Tamotsu Kuroki.

**Visualization:** Hiroaki Takeshita, Kentaro Hara, Kodai Ichihara, Michiko Yamaguchi, Tamotsu Kuroki.

**Writing – original draft:** Hiroaki Takeshita, Kentaro Hara, Kodai Ichihara, Michiko Yamaguchi, Tamotsu Kuroki.

**Writing – review & editing:** Kentaro Hara, Hiroaki Takeshita, Kodai Ichihara, Michiko Yamaguchi, Tamotsu Kuroki.
